# Outcomes with mismatched unrelated donor allogeneic hematopoietic stem cell transplantation in adults: A systematic review and meta-analysis

**DOI:** 10.3389/fonc.2022.1005042

**Published:** 2022-10-06

**Authors:** Muhammad Umair Mushtaq, Moazzam Shahzad, Ezza Tariq, Qamar Iqbal, Sibgha Gull Chaudhary, Muhammad U. Zafar, Iqra Anwar, Nausheen Ahmed, Rajat Bansal, Anurag K. Singh, Sunil H. Abhyankar, Natalie S. Callander, Peiman Hematti, Joseph P. McGuirk

**Affiliations:** ^1^ Division of Hematologic Malignancies and Cellular Therapeutics, University of Kansas Medical Center, Kansas City, KS, United States; ^2^ Moffitt Cancer Center, University of South Florida, Tampa, FL, United States; ^3^ Department of Medicine, University of Toledo Medical Center, Toledo, OH, United States; ^4^ School of Medicine and Public Health, University of Wisconsin, Madison, WI, United States

**Keywords:** mismatched unrelated donor, allogeneic hematopoietic stem cell transplantation, outcomes, hematologic malignancies, donor selection, HLA matching in bone marrow transplantation

## Abstract

**Background:**

Allogeneic hematopoietic stem cell transplantation (HSCT) is a potentially curative therapy for various hematologic disorders. Alternative donor strategies such as mismatched unrelated donors (MMUD) offer the option of HSCT to patients lacking a human leukocyte antigen (HLA)-matched donor. We conducted a systematic review and meta-analysis to evaluate outcomes after MMUD-HSCT.

**Methods:**

A literature search was performed on PubMed, Cochrane Library, and ClinicalTrials.gov from the inception date through April 6, 2022. After screening 2477 manuscripts, 19 studies were included. Data was extracted following the Preferred Reporting Items for Systematic Reviews and Meta-Analysis (PRISMA) guidelines. Pooled analysis was done using the meta-package by Schwarzer et al. Proportions with 95% confidence intervals (CI) were computed.

**Results:**

A total of 3336 patients from 19 studies were included. The median age was *52.1* years, and 53% of recipients were males. The graft source was bone marrow in 19% and peripheral blood stem cells in 81% of recipients. The median time to transplant from hematologic diagnosis was 10 (1-247) months. Hematologic diagnoses included myeloid (82.9%), lymphoid (41.1%), and other disorders (3%). The reduced intensity and myeloablative conditioning were used in 65.6% and 32% of recipients, respectively. *In-vivo* T-cell depletion was performed in 56.7% of the patients. Most patients had one (87.9%) or two (11.4%) antigen HLA-mismatch. The pooled 1-year overall survival (OS) was 63.9% (95% CI 0.57-0.71, n=1426/2706), and the pooled 3-year OS was 42.1% (95% CI 0.34.2-0.50, n=907/2355). The pooled progression-free survival was 46.6% (95% CI 0.39-0.55, n=1295/3253) after a median follow-up of 1.8 (range 1-6) years. The pooled relapse rate was 26.8% (95% CI 0.22-0.32, n=972/3253) after a median follow-up of 2.25 (1-3) years. The pooled incidence of acute (grade II-IV) graft-versus-host disease (GVHD) and chronic GVHD was 36.4% (95% CI 0.31-0.42, n=1131/3030) and 41.2% (95% CI 0.35-0.48, n=1337/3228), respectively. The pooled non-relapse mortality was 22.6% (95% CI 0.17-0.29, n=888/3196) after a median follow-up of 2.6 (1-5) years.

**Conclusion:**

MMUD-HSCT has demonstrated favorable outcomes with an acceptable toxicity profile. It represents a promising option in patients lacking an HLA-matched or haploidentical donor and may expand HSCT access to underrepresented racial and ethnic populations.

## Introduction

Allogeneic hematopoietic stem cell transplantation (HSCT) is a potentially curative therapy for various high-risk hematologic malignancies and non-malignant disorders. Almost half of stem cell transplants performed in the United States are allogeneic (1). The first human leukocyte antigen (HLA) was discovered in 1958, and it was quickly found that HLA mismatch plays a significant role in the development of graft-versus-host disease (GVHD) and non-relapse mortality (NRM) among transplant recipients (2). Outcomes of HSCT have improved with the emphasis on HLA matching. The first successful HSCT utilized a related donor. The matched sibling donor (MSD) remains an ideal source of stem cell transplantation; however, the chance of finding an MSD is only 25-30% (3, 4). The worldwide donor registries, including Bone Marrow Donors Worldwide (BMDW)/World Marrow Donor Association (WMDA) and National Marrow Donor Program (NMDP), were established to improve the chances of finding a non-related HLA-matched donor (4, 5). There is a significant over-representation of Caucasian donors in these registries, with a paucity of donors for many racial and ethnic groups (6, 7). Since 2014, the chances of finding a matched donor improved from 25% to 75% for Caucasians, 7% to 29% for African-Americans, and 10% to 48% for Hispanics (6). The racial disparity in access to the matched unrelated donor is multifactorial, including frequent HLA heterogeneity, lower representation in donor registries, and lack of access to health care and education (4, 8–10).

Patients who do not have an HLA-matched donor can still potentially undergo transplantation by strategies aimed to cross HLA barriers, including utilizing an alternative donor source, such as haploidentical (haplo) family donors, mismatched unrelated donors (MMUD), or umbilical cord blood cells (UCB), and improved GVHD prophylaxis using interventions such as T-cell depletion (TCD) and post-transplant cyclophosphamide (PT-Cy) (11, 12). These strategies also have improved access to HSCT for currently underrepresented populations in donor registries (13, 14). We conducted this systematic review and meta-analysis to investigate the outcomes following MMUD HSCT.

## Methods

### Data sources and search strategy

The literature search for the systematic review and meta-analysis was carried out following the guidelines outlined in the Preferred Reporting Items for Systematic Reviews and Meta-Analysis (PRISMA) (15). Population, intervention, comparison, and outcome (PICO) tables were developed. Three electronic databases (PubMed, Cochrane Library, and ClinicalTrials.gov) were searched thoroughly through April 6, 2022, using the MeSH terms and entry words for “hematopoietic stem cell transplantation” “hematologic neoplasms,” unrelated donors,” and “treatment outcome.” No filters or publication time limits were applied for the search, and 2480 records were identified. A manual search of the professional meeting abstracts, e.g., the American Society of Hematology and the American Society of Clinical Oncology, identified two records. All search results were imported into the Endnote X9.0 reference manager, and duplicates were removed.

### Selection criteria

**Figure 1:**
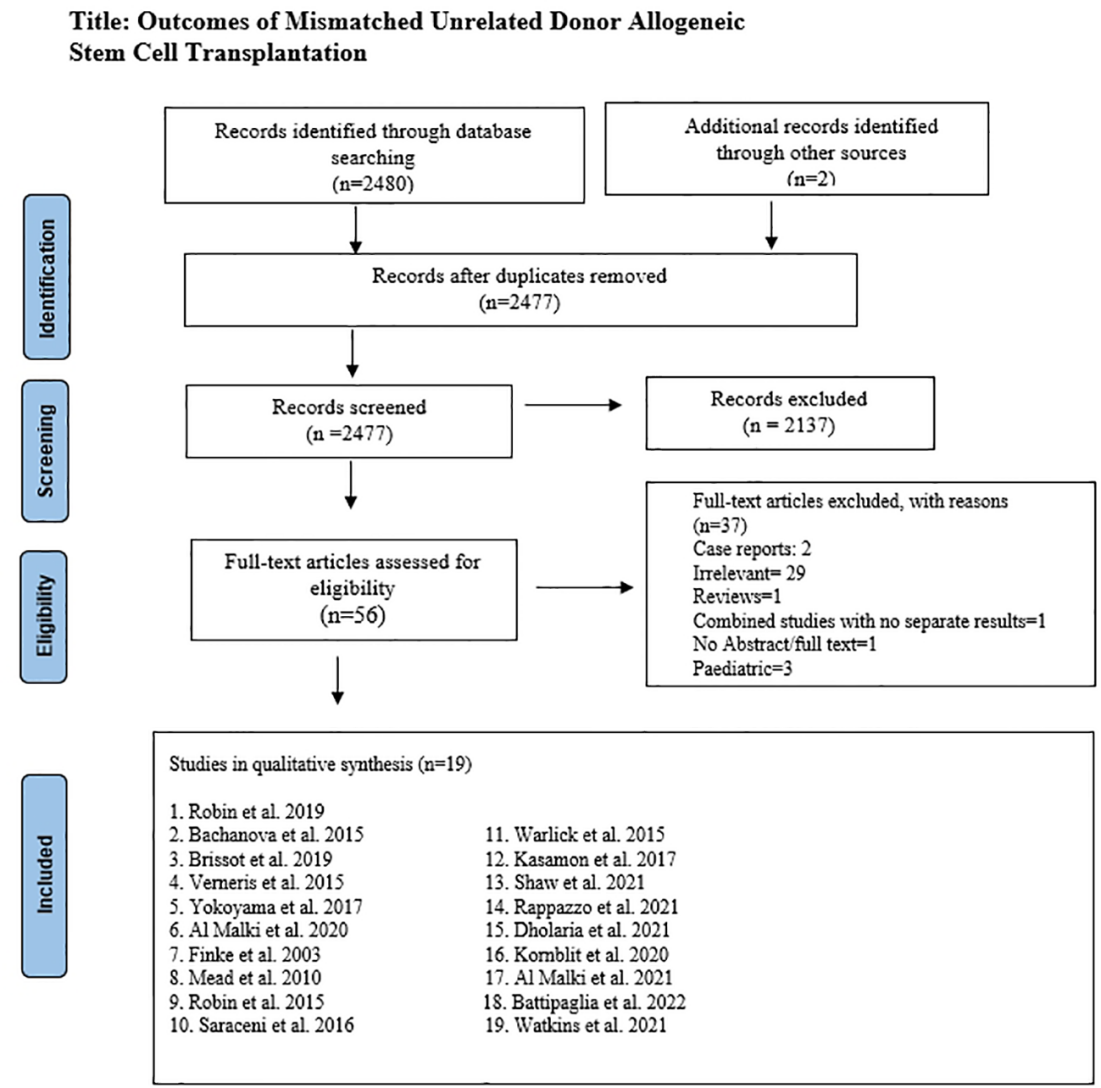
PRISMA Diagram for included and excluded studies.

After removing duplicates, 2477 articles were screened by two authors independently. In primary screening, non-relevant articles were excluded based on title and abstract. Full texts of the remaining 56 articles were then assessed for eligibility based on predetermined criteria, set after discussion and consensus between all authors, and approved by the principal investigator (M.U.M.). Inclusion criteria were original studies (clinical trials, case-control, retrospective, and prospective cohort) that reported outcomes with mismatched unrelated donor HSCT. Only adult studies were included in the analysis except Watkins et al. (16), which had few pediatric patients. This study was included because of its clinical significance and most of the patients were adults. A total of 37 studies were excluded in secondary screening based on relevancy, case reports, pediatric population, non-availability of the abstract or full-length article, and articles in a language other than English. (**Figure 1**) **Supplementary Table S1** lists excluded studies and the reasons for exclusion.

### Data extraction

Two authors extracted data from the 19 selected studies independently. Following individual data extraction, data sheets were double-checked for any discrepancies. In addition, Data was collected for baseline characteristics, including the number of patients, gender, age, indication for HSCT/hematologic diagnosis, HSCT platform, source of stem cells for primary graft, HLA status of the donor, and efficacy and safety, including overall survival (OS), progression-free survival (PFS), relapse rate (RR), acute and chronic GVHD, and NRM.

### Quality evaluation

The methodological quality of the included studies was evaluated using the National Institute of Health (NIH) quality assessment tool. The Joanna Briggs Institute (JBI) Critical Appraisal Checklist for Studies Reporting Prevalence Data was used for quality assessment, and all studies were reported as good.

### Data analysis

Proportions along with 95% confidence intervals (CIs) were extracted to compute pooled analysis using the R ‘meta-package in R version 4.16-2 (R Foundation for Statistical Computing, Vienna, Austria) (17). We pooled the experimental arm results of included trials using the inverse variance method and logit transformation. The variance among studies was calculated using the Der Simonian-Laird estimator (18).

## Results

A total of 3336 patients from 19 studies were included in this systematic review and meta-analysis, with a median age of 52.1 (6-76.5) years (16, 19–36), and 53% (n=1659/3116) of recipients were male (20–25, 27–36). Eighty-six percent (n=1055/1222) patients were Caucasian and 5.8% (n=71/1222) were African American (30–16). The primary graft source was bone marrow (BM) in 19% (n=646/3336) and peripheral blood stem cells (PBSC) in 81% (n=2690/3336) of the recipients (16, 19–36). The median time to transplant was 10 (1-247) months (15, 20, 22–24, 28) and median follow-up time was 33 (0.4-135.6) months (16, 20–22, 24–32, 34–37). Reduced-intensity conditioning (RIC) conditioning was used for 65.6% (n=2191) of the patients while 32% (n=1066) of patients received myeloablative conditioning (MAC). *In-vivo* TCD was performed in 56.7% of the patients, as reported by ten studies with available data (n=1359/2395) (20–26, 28, 30, 31). Among them, 20% (n=272) patients received anti-thymocyte globulin (ATG) and 9.2% (n=125) patients received alemtuzumab whereas details regarding *in-vivo* TCD regimen were not available in 70.8% (n=962) of patients from studies based on registry databases. Underlying hematologic diagnoses included myeloid disorders (82.9%, n=2767/3336), lymphoid disorders (41.1%, n=471/3336), and others (3%, n=98/3336). HLA mismatch (MM) status was reported as HLA 9/10 MM (62%, n=1462/2360), HLA 7/8 MM (26%, n=612/2360), HLA 8/10 MM (5.7%, n=136/2360), HLA 6/8 MM (5.7%, n=134/2360), and 3 or higher HLA MM (0.7%, n=16/2360) of the patients (19, 21–23, 28, 34). Six studies (n=1068) specified the type of allele mismatch (MM) as follows: HLA-A MM in 26.3% (n=281/1068), HLA-B MM in 11.9% (n=127/1068), HLA-C MM in 34.2% (n=366/1068), HLA-DRB1 MM in 11.1% (n=119/1068), HLA-DQB1 MM in 17.3% (n=185/1068), and HLA-DPB1 MM in 29.6% (n=317/1068) of the patients (21, 25, 30, 31, 34, 35) (**Table 1**)

**Table 1:** Baseline characteristics of patients with mismatched unrelated donor allogeneic stem cell transplantation.

Author, Year	Pts,n	Age, median yrs (range)	Male,n (%)	Race, n (%)	Time to transplant, median mo (range) ^a^	Graft source		Underlying hematological disorders			HLA Matching,n (%)	HLA Loci Mismatch					
						BMn (%)	PBSCn (%)	Myeloid,n (%)	Lymphoid,n (%)	Other,n (%)		HLA-An (%)	HLA-Bn (%)	HLA-C n (%)	HLA-DRB1n (%)	HLA-DQB1n (%)	HLA-DPB1n (%)
**Robin et al., 2019**	443	59 (52-65)	277 (62.5)	NA	11 (7-21)	43 (10)	400 (90)	367 (83)	0 (0)	76 (17)	NA	NA	NA	NA	NA	NA	NA
**Bachanova et al., 2015**	275	45 (18-71)	164 (60)	C: 246 (89), AA: 16 (6), O: 13 (5)	32 (3–247)	74 (27)	201 (73)	NA	275 (100)	0 (0)	NA	78 (28)	38 (14)	130 (47)	29 (11)	NA	NA
**Brissot** **et al. 2019^b^**	383	51.7 (18-76)	209 (54.6)	NA	8 (2-121)	30 (8)	353 (92)	383 (100)	0 (0)	0 (0)	HLA 9/10: 383 (100)	NA	NA	NA	NA	NA	NA
**Verneris** **et al. 2015**	563	58 (19-76)	306 (54.4)	C: 508 (90), AA: 30 (5), O: 25 (5)	NA	90 (16)	473 (84)	519 (92)	44 (8)	0 (0)	HLA 7/8: 563 (100)	188 (33)	81 (14)	219 (39)	75 (13)	SA-M: 36 (6) DA-M: 1 (<1)	SA-M: 175(31) DA-M: 110(20)
**Yokoyama et al., 2017**	115	57 (18-68)	NA	NA	NA	115 (100)	0 (0)	98 (85)	17 (15)	0 (0)	HLA 6/8: 115 (100)	NA	NA	NA	NA	NA	NA
**Al Malki** **et al. 2020**	131	45 (18-71)	80 (61.1)	NA	NA	0 (0)	131 (100)	73 (56)	51 (39)	7 (5)	NA	NA	NA	NA	NA	SA-M 14(11) DA-M 117(89)	NA
**Finke et al., 2003**	25	37 (23-56)	NA	NA	NA	23 (92)	2 (8)	21 (84)	4 (16)	0 (0)	NA	NA	NA	NA	NA	NA	NA
**Mead et al., 2010**	50	48 (18-67)	28 (56)	NA	NA	16 (32)	34 (68)	15 (30)	29 (58)	6 (12)	NA	5 (10)	0 (0)	13 (26)	5 (10)	9 (18)	NA
**Robin et al., 2015^b^**	107	61 (20-74)	69 (65)	NA	10 (1.6-141)	0 (0)	107 (100)	107 (100)	0 (0)	0 (0)	HLA 9/10: 107 (100)	NA	NA	NA	NA	NA	NA
**Saraceni et al., 2016**	375	49 (18-69)	188 (50)	NA	6 (2.7–25.5)	58 (16)	317 (84)	375 (100)	0 (0)	0 (0)	HLA 9/10: 375 (100)	NA	NA	NA	NA	NA	NA
**Warlick et al., 2015**	21	NA	14 (67)	NA	14.4 (4-164)	9 (43)	12 (57)	21 (100)	0 (0)	0 (0)	NA	NA	NA	NA	NA	NA	NA
**Kasamon et al., 2017**	20	56 (37-66)	12 (60)	C: 17 (85), AA: 3 (15)	NA	20 (100)	0 (0)	16 (80)	4 (20)	0 (0)	NA	NA	NA	10 (50)	NA	NA	9 (45)
**Shaw et al., 2021**	80	51.5 (18-70)	42 (53)	C: 60 (75), AA: 15 (19), O: 5 (6)	NA	80 (100)	0 (0)	61 (76)	19 (24)	0 (0)	HLA 7/8: 49 (61), HLA 6/8: 19 (24), HLA: 5/8 7 (9), HLA: 4/8 5 (6)	NA	NA	NA	NA	NA	NA
**Rappazzo et al., 2021**	29	54 (22-74)	14 (48)	NA	NA	0 (0)	29 (100)	19 (65)	9 (31)	1 (4)	HLA 9/10: 19 (66), HLA 8/10: 6 (21), HLA 7/10: 4 (14)	10 (34)	8 (28)	4 (14)	10 (34)	8 (28)	23 (79)
**Dholaria et al., 2021**	280	52.1 (18.2-75.6)	163 (58)	NA	NA	19 (6.8)	261 (93.2)	280 (100)	0 (0)	0 (0)	HLA 9/10: 280 (100)	107 (38)	56 (20)	52 (19)	23 (8)	42 (15)	NA
**Kornblit et al., 2020**	76	63 (55-67)	49 (64)	C: 65 (86), AA: 3 (4), O: 8 (10)	NA	0	76 (100)	57 (74)	14 (18)	5 (7)	HLA 8/10: 76 (100); class I MM 51 (67), class II MM 25 (33)	NA	NA	NA	NA	NA	NA
**Al Malki et al., 2021**	38	53 (21-72)	19 (50)	C: 24 (63), AA: 4 (11), O: 10 (26)	NA	0 (0)	38 (100)	35 (92)	3 (8)	0 (0)	HLA 8/10: 38 (100); class I MM 33 (87), class II MM 30 (79), DQ MM 2 (5)	15 (39.5)	12 (31.6)	8 (21)	5 (13.2)	NA	NA
**Battipaglia et al., 2022**	155	52 (18-76)	67 (43)	NA	6 (1-86)	0 (0)	155 (100)	155 (100)	0 (0)	0 (0)	HLA 9/10: 155 (100)	55 (35.5)	35 (28.6)	29 (18.7)	13 (8.4)	23 (14.8)	NA
**Watkins et al., 2021**	43 (ITT)	39 (6.6-76.5)	NA	C: 30 (70)	NA	21 (49)	22 (51)	39 (91)	1 (2)	3 (7)	HLA 9/10: 39 (90.7), HLA 8/10: 4 (9)	NA	NA	NA	NA	NA	NA
	127 (Control arm)	45 (6-74.4)	NA	C: 105 (82.7)	NA	48 (38)	79 (62)	126 (99)	1 (1)	0 (0)	HLA 9/10: 104 (90), HLA 8/10: 12 (10)	NA	NA	NA	NA	NA	NA

a Time from diagnosis to transplant.

Pts, patients; Yrs, years; Mo, months; BM, Bone marrow; PBSC, Peripheral blood stem cell transplant; GVHD, Graft versus host disease; HLA, human leukocyte antigen; C, Caucasian; AA, African-American; O, Others; NA, not available; CYA, Cyclosporine; MMF, mycophenolate mofetil; MTX, Methotrexate; TAC, tacrolimus; CP, cyclophosphamide; Siro, Sirolimus; ATG, Anti-thymocyte globulin; CS, corticosteroids; NHL, non-Hodgkin lymphoma; HL, Hodgkin lymphoma; MMUD, mismatch unrelated donor; HLA, Human leukocyte antigen; SA-M, Single-allele mismatch; DA-M, Double-allele mismatch; ITT, Intent to treat.

**Figure 2:**
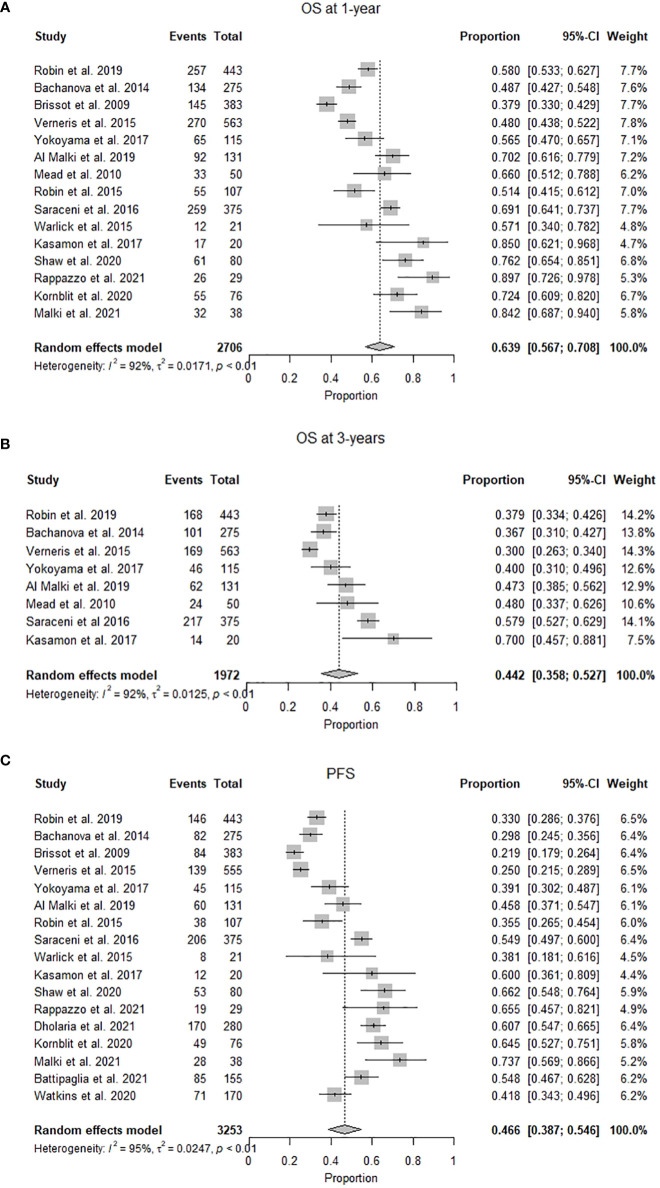
**(A)** Forest plot of pooled 1-year overall survival after mismatched unrelated donor allogeneic stem cell transplant (n=2706). **(B)** Forest plot of pooled 3-year overall survival after mismatched unrelated donor allogeneic stem cell transplant (n=1972). **(C)** Forest plot of pooled progression-free survival after mismatched unrelated donor allogeneic stem cell transplant (n=3253).

**Figure 3:**
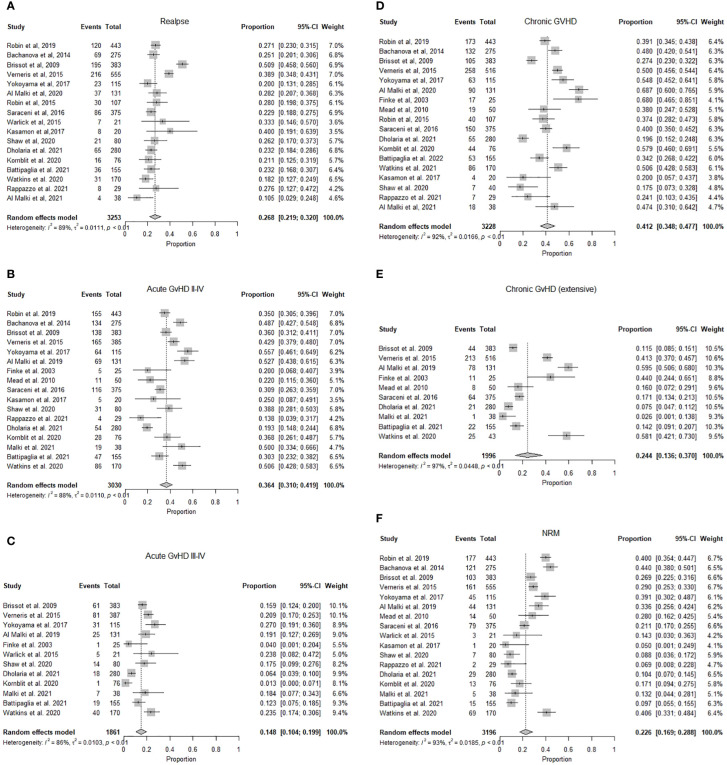
**(A)** Forest plot of pooled incidence of relapse after mismatched unrelated donor allogeneic stem cell transplant (n=3253). **(B)** Forest plot of pooled incidence of acute graft versus host disease grade (II-IV) after mismatched unrelated donor allogeneic stem cell transplant (n=3030). **(C)** Forest plot of pooled incidence of acute graft versus host disease grade (III-IV) after mismatched unrelated donor allogeneic stem cell transplant (n=1861). **(D)** Forest plot of pooled incidence of chronic graft versus host disease after mismatched unrelated donor allogeneic stem cell transplant (n=3228). **(E)** Forest plot of pooled incidence of chronic graft versus host disease (extensive) after mismatched unrelated donor allogeneic stem cell transplant (n =1996). **(F)** Forest plot of pooled incidence of non-relapse mortality after mismatched unrelated donor allogeneic stem cell transplant (n=3196).

OS ranged from 93% at one year to 28% at three years. The pooled OS was 63.9% (95% CI 0.57-0.71, I^2^= 92%, n=1426/2706) at one year, while the pooled OS at three years was 42.1% (95% CI 0.34.2-0.50, I^2^ = 93%, n=1513/2355). (**Figures 2A, B**) The pooled PFS was 46.6% (95% CI 0.39-0.55, I^2^ = 95% n=1295/3253) at a median follow-up of 1.8 (1-6) years. (**Figure 2C**) The pooled RR was 26.8% (95% CI 0.22-0.32, I^2^ = 89%, n=972/3253) at a median follow-up of 2.25 (1-3) years. (**Figure 3A**) The pooled incidence of acute GVHD (grade II-IV) was 36.4% (95% CI 0.31-0.42, I^2^ = 88%, n=1131/3030) and the pooled incidence of acute GVHD (grade III-IV) was 14.8% (95% CI 0.10-0.19, I^2^ = 86%, n=369/1861). (**Figures 3B, C**) The pooled incidence of chronic GVHD was 41.2% (95% CI 0.35-0.48, I^2^ = 93%, n=1337/3228). The pooled incidence of chronic GVHD (extensive) was 24% (95% CI 0.14-0.37, I^2^ = 92%, n=487/1996). (**Figures 3D, E**) At a median follow-up of 2.6 (1-5) years, the pooled NRM was 22.6% (95% CI 0.17-0.29, I^2^ = 93% n=888/3196) (**Figure 3F** and **Table 2**).

**Table 2:** Outcomes of mismatched unrelated donor allogeneic stem cell transplantation.

Author, Year	Pts, n	Conditioning,n (%)	GVHD prophylaxis,n (%)	T Cell Depletion, n (%)	Median follow-up, mo (range)	1-yr OS,n (%)	3-yr OS,n (%)	PFS,n (%)	Relapse,n (%)	Acute GvHD II-IV, n (%)	Acute GvHD III-IV, n (%)	Chronic GVHD n (%)		NRM,n (%)
												All grades	Extensive	
**Robin et al., 2019**	443	MAC: 134(30) RIC: 308 (70)	NA	*In-vivo* TCD: 381 (86)	33	257 (58)	168 (38)	146 (33)	120 (27)	155 (35)	NA	173 (39)	NA	177 (40)
**Bachanova et al., 2015**	275	MAC: 81 (29) RIC: 194 (71)^1^	Tac + O: 179 (65), CYA + O: 43 (16), O: 4 (3)	ATG: 88 (32) ALZ: 38 (14) ATG+ALZ: 1 None: 148 (54)	65 (12-125)	134 (49)	101 (37)	82 (30)	69 (25)	134 (49)^a^	66 (24)^a^	132 (48)	NA	121 (44)
**Brissot et al., 2019^b^**	383	MAC: 144 (38) RIC: 238 (62)^2^	CYA + MTX: 113 (30), CYA + MMF: 161 (42), Tac + MMF: 27 (7), CYA + MMF + MTX: 11 (3), CP: 7 (2)	*In-vivo* TCD: 327 (85.8)	22.9 (1.8-104.9)	145 (38)	106 (28)^b^	84 (22)	195 (51)	138 (36)	61 (16)	105 (27)	44 (11.6)	103 (27)
**Verneris et al., 2015**	563	RIC: 563 (100)^3^	TAC + O: 392 (70), CYA + O: 158 (28)	*In-vivo* TCD: 253 (45)	48 (3-125)	270/563 (48)	169/563 (30)	139/555 (25)	216/555 (39)	165/385 (43)^a^	81/387 (21)^a^	258/516 (50)	213 (38)	161/555 (29)
**Yokoyama et al., 2017**	115	RIC: 115 (100)^4^	CYA: 16 (14), Tac: 94 (82)	None	NA	65 (57)	46 (40)	45 (39)	23 (20)	64 (56)^a^	31 (27)^a^	63 (55)	NA	45 (39)
**Al Malki et al, 2020**	131	MAC: 71 (54) RIC: 60 (46)^5^	CNI: 35 (27), CNI + MTX: 77 (59), CNI + ATG: 19 (14)	ATG: 19 (14)	74.4 (28.8-135.6)	92 (70)	62 (48)	60 (46)	37(28)	69 (53)^a^	25 (19)^a^	90 (69)	78 (59.5)	44 (34)^e^
**Finke et al., 2003**	25	MAC: 20 (80) RIC: 5 (20)^6^	ATG: 25 (100)	ATG: 25 (100)	35.1 (0.4 - 64.4)	NA	NA	NA	NA	5 (20)^a^	1 (4)^a^	17 (67)^a^	11 (44)	NA
**Mead et al., 2010**	50	RIC: 50 (100)^7^	NA	ALZ: 50 (100)	27.6	33 (66)	24.5 (49)	NA	NA	11 (22)	NA	19 (39)^c^	8 (15)^c^	14 (27)
**Robin et al., 2015^b^**	107	RIC: 107 (100)^8^	CYA + MMF: 56 (52), CYA + MTX: 15 (14), CYA +/- CS: 28 (26), Others: 8 (8)	ATG: 64 (69) ALZ: 29 (31)	NA	55 (51)	46 (43)^b^	38 (36)	30 (28)	NA	NA	40 (37)	NA	NA
**Saraceni et al., 2016**	375	MAC: 194 (52) RIC: 180 (48)	NA	NA	25 (1-113)	259 (69)	217 (58)	206 (55)	86 (23)	116 (31)	NA	150 (40)^b^	64 (17)	79 (21)
**Warlick et al., 2015**	21	MAC: 19 (90) RIC: 2 (10)^9^	CYA + MMF: 2 (10), CYA + MTX: 19 (90)	ATG: 10 (48)	62.4	12.4 (59)	11 (51)^d^	8 (39)^d^	7 (33)^b^	NA	5 (24)^a^	NA	NA	3 (14)
**Kasamon et al., 2017**	20	RIC: 20 (100)^10^	PT-Cy + MMF + Sir or Tac: 20 (100)	None	48	17 (75)	14(62)	12 (52)	8 (35)^c^	5 (20)^a^	NA	4 (16)^c^	NA	1 (6)^b^
**Shaw et al., 2021^c^**	80	MAC: 40 (50)	PT-Cy + MMF + Sir: 80 (100)	None	12 (5.4-12)	61 (76); MAC: 29 (72.5), RIC: 32 (80)	NA	53 (67); MAC: 25 (61.2), RIC: 27 (67.5)	21 (26); MAC: 12 (30), 9 (23) ^c^	30 (37.5); 17 (43), 13 (33) ^a^	7 (8.7); 7 (18), (0) ^a^	21 (26); 14 (36), 7 (18) ^c^	NA	7 (8.7); 3 (8), 4 (10) ^c^
		RIC: 40 (50)^11^												
**Rappazzo et al., 2021**	29	RIC: 29(100)^12^	PT-Cy + MMF + Sir: 29 (100)	None	8	26 (93)	NA	19 (64)^c^	8 (29)^c^	4 (15)	NA	7 (23)	NA	2 (7)^c^
**Dholaria et al., 2021**	280	MAC: 141(50.4) RIC: 139 (49.6)	CYA + MMF: 111 (39.6), Tac + MMF 51 (18.2), CYA + MTX:10(3.6), CYA: 34(12.1), Tac: 24(8.6), Siro + MMF:14(5), Other: 36 (12.9)	ATG: 66 (23.5) ALZ: 8 (2.8)	19.1 (11.4-36.7)	NA	117 (66.1)^b^	170 (60.5) ^b^	65 (23.2) ^b^	54 (31.3) ^f^	18 (10.5)	55 (32.6) ^b^	21 (12.6) ^b^	29 (16.7) ^b^
**Kornblit et al., 2020**	76	NMA: 76 (100) ^g^	Siro + CYA + MMF: 76 (100)	NA	47 (4-94)	55 (72)	NA	49 (64) ^c^	16 (21) ^c^	28 (36) ^a^	1 (1.3)	44 (57) ^h^	NA	13 (18) ^h^
**Al Malki et al., 2021**	38	MAC: 19 (50) RIC: 19 (50)	PTCy + MMF + Tac: 38 (100)	NA	18.3 (8.7-25)	32 (84)	NA	28 (76)	4 (11)^c^	19 (50) ^a^	7 (18) ^a^	18 (49)	1 (3)	5 (13) ^c^
**Battipaglia et al., 2022**	155	MAC: 83 (54) RIC: 72 (46)	CNI: 33 (21), CNI + MMF 112 (72), MMF: 5 (3), MTX: 1 (1), PTCy: 2 (1.5)	NA	1.9 (1.3-2.1)	NA	118 (76)^b^	85 (67) ^b^	36 (23) ^b^	47 (30) ^a^	19 (12) ^a^	53 (34) ^b^	22 (14) ^b^	15 (10) ^b^
**Watkins et al., 2021**	43 (ITT)	MAC: 33 (77) RIC: 10 (23)	Tac: 29 (67) or CYA: 14 (33) + MTX 43 (100) + abatacept 46 (100)	NA	59 (NA)	NA	32 (73.6)^b^	32 (74) ^b^	4 (9.3) ^b^	18 (41.9) ^a^	1 (2.3) ^a^	27 (62) ^c^	25 (57.9) ^c,i^	7 (16.7) ^b^
	127 (Control arm)	MAC: 87 (68) RIC: 40 (32)	Tac: 96 (76) or CYA: 31 (24) + MTX 127(100)	NA	59 (NA)	NA	58 (45.3)^b^	49 (38.3) ^b^	27 (21.4) ^b^	68 (53.2) ^a^	39 (30.2) ^a^	59 (45.9) ^c^	NA	52 (40.3) ^b^

NA, not available; Pts, patients; Yr, year; OS, overall survival; PFS, Progression-Free Survival; aGVHD, acute graft versus host disease; cGVHD, chronic graft versus host disease; NRM, Non-relapse mortality; MAC, myeloablative conditioning; RIC, reduced intensity conditioning; Flu, Fludarabine; BU, busulfan; TBI, total body irradiation; Mel, melphalan; Cy, cyclophosphamide; PT, post-transplant; CYA, cyclosporine A; ATG, Anti-thymocyte globulin; ALZ, Alemtuzumab; MMF, mycophenolate mofetil; CNI, calcineurin inhibitor; Tac, tacrolimus; Sir, sirolimus; MTX, methotrexate; ITT, intent to treat.

All outcomes are at 3 years unless otherwise mentioned. ^a^outcome at 100 days, ^b^outcomes at 2 years, ^c^outcomes at 1 year, ^d^outcomes at 6 years, ^e^outcomes at 5 years. ^f^outcome at 6 months. ^g^NMA: Fludarabine 90mg/m^2^ and 2-3 Gy total body irradiation. ^h^ outcomes at 4 years. ^i^moderate-severe cGVHD,

^1^RIC: Mel <140mg/m^2^, BU <9 mg/kg, TBI <5 Gy, Flu/TBI; MAC: TBI or BU based combinations. ^2^MAC: TBI >6 Gy, BU (oral) >8mg/kg, BU (IV) >6.4mg/kg; RIC: Flu 30 mg/m^2^/day, CYA 2g/m^2^/day, amsacrine 100mg/m2/day, TBI 4 Gy, Cy 40-60 mg/kg/day, IV BU 6.4 mg/kg. ^3^RIC regimen was defined as opposite of MAC. MAC: TBI >5 Gy or >8 Gy total in fractionated doses, Bu > 9 mg/kg, Mel > 150 mg/m^2^, or thiotepa > 10 mg/kg. ^4^RIC: TBI ≤8 Gy, BU <9 mg/kg, or Mel ≤140 mg/m^2^. ^5^MAC: TBI >5 Gy or >8 Gy in fractionated doses, BU >9 mg/kg, or Mel >150 mg/m^2^, RIC defined as others. ^6^MAC: TBI 12 Gy, Etoposide 50 mg/kg, Cy 60 mg/kg; RIC: Flu, Mel 110 mg/m2, carmustine 300 mg/m^2^. ^7^ALZ 20 mg/d, Flu 30 mg/m^2^/d, Mel 140 mg/m2/d, CYA. ^8^RIC: Flu/Mel, FluBu, FluTBI,FluCy 2 Gy TBI. ^9^MAC: Cy 120 mg/kg & BU 3.2 mg/kg, or 12 Gy TBI fractionated or Cy 60 mg/kg+ TBI 165 cGy or Flu 25 mg/m2 Cy TBI; RIC: Flu 30 mg/m2, BU 3.2 mg/kg plus rabbit ATG OR Cy 50mg/kg + Flu + TBI. ^10^Cy 14.5 mg/kg, Flu 30 mg/m2, TBI 2 Gy. ^11^MAC: Cy+ TBI or Cy+ BU or Flu+ BU; RIC: Flu+ Cy+ low dose TBI. ^12^Cy 14.5mg/kg, Flu 20 mg/m2, TBI 2 Gy.

### Outcomes after MMUD HSCT without post-transplant cyclophosphamide for GVHD prophylaxis

Fifteen studies, including 3169 patients, did not use PT-Cy for GVHD prophylaxis. The median age was 51.7 (6-76.5) years (16, 19–32), and 56.5% (n=1614/2859) recipients were male (20–25, 27–32). The graft source was BM in 17% (n=546/3169) and PBSC in 82% (n=2623/3169) of the recipients (16, 19–32). The median time to transplant from hematologic diagnosis was 10 (1-247) months (20, 22–24, 28, 30), and median follow-up time was 35.1 (0.4-135.6) months (16, 20–32). The pooled OS was 57.5% (95% CI 0.50-0.64, I^2^ = 92%, n=1377/2539) at one year (19–25, 28, 30–32), while the pooled OS at three years was 44.2% (95% CI 0.35.2-0.52, I^2^ = 92%, n=787/1952) (19, 21, 22, 24, 25, 28, 30, 31). The pooled PFS was 41.5% (95% CI 0.33-0.49 I^2^ = 95% n=1183/3086) at a median follow-up of 2.7 (1-6) years (16, 19–24, 27–32). The pooled RR was 27.4% (95% CI 0.22-0.33, I^2^ = 91%, n=931/3086) at a median follow-up of 2.5 (1-3) years (16, 19–24, 27–32). The pooled incidence of acute GVHD (grade II-IV) was 37.3% (95% CI 0.31-0.43, I^2^ = 90%, n=1072/2863) (19, 21–23, 25–32), and the pooled incidence of acute GVHD (grade III-IV) was 15.2% (95% CI 0.10-0.20, I^2^ = 89%, n=369/2018 (16, 19–21, 26–32). The pooled incidence of chronic GVHD was 44.3% (95% CI 0.37-0.51, I^2^ = 93%, n=1337/3101) (16, 19, 21–32). The pooled incidence of chronic GVHD (extensive) was 27.4% (95% CI 0.15.7-0.41, I^2^ = 97%, n=486/1958) (16, 21, 22, 25, 26, 28, 29, 31). At a median follow-up of 3 (1-5) years, the pooled NRM was 26.3% (95% CI 0.20-0.33, I^2^ = 93% n=863/3029) (16, 19–22, 24, 25, 27–31) (**Tables 1**, **2**).

### Outcomes after MMUD HSCT with post-transplant cyclophosphamide for GVHD prophylaxis

Four studies, including 167 patients, reported the use of PT-Cy for GVHD prophylaxis. The median age was 54.2 (18-76) years, and 26.9% (n= 45/167) were male (33–36). The pooled OS was 81.9% (95% CI 0.75-0.88, I^2^ = 0%, n=136/167) at one year (33–36). At a median follow-up of 1.8 (1-6) years, the pooled PFS was 67.3% (95% CI 0.6-0.74, I^2^ = 0% n=112/167) (33–36). The pooled RR was 24.3% (95% CI 0.14-0.36, I^2^ = 89%, n=41/167) at a median follow-up of 2.25 (1-3) years (33–36). The pooled incidence of acute GVHD (grade II-IV) was 32.1% (95% CI 0.18-0.48, I^2^ = 74%, n=59/167) (33–36), and the pooled incidence of acute GVHD (grade III-IV) was 17.7% (95% CI 0.11-0.25, I^2^ = 0%, n=21/118) (33–36). The pooled incidence of chronic GVHD was 30.5% (95% CI 0.20-0.42, I^2^ = 51%, n=52/167) (33–36). At a median follow-up of 2.6 (1-5) years, the pooled NRM was 8.6% (95% CI 0.04-0.14, I^2^ = 93% n=15/167) (33–36) (**Tables 1** and **2**).

### Outcomes after MMUD HSCT using reduced intensity conditioning

Seven studies, including 924 patients, reported RIC conditioning with a median age of 57.5 (18-76) years (19, 21, 23, 25, 34, 35, 37). Fifty-six percent (n= 429/769) recipients were males (21, 23, 25, 33–35). The graft source was PBSC in 69.6% (n=643/924) and BM in 30.4% (n=281/924) of the patients (19, 21, 23, 25, 34, 35, 37). The median follow-up time was 28 (3-125) months. The pooled 1-year was 66.9% (95% CI 0.55-0.77, I^2^ = 88%, n=498/924) (19, 21, 23, 25, 34, 35, 37), and 3-years OS was 43.5% (95% CI 0.33-0.54, I^2^ = 84% n=300/855) (19, 21, 23, 25, 35). The pooled incidence of acute GVHD (grade II-IV) was 33% (95% CI 0.22-0.45, I^2^ = 84%, n=262/639) (19, 22, 25, 33–35), while the pooled incidence of acute GVHD (grade III-IV) was 16.4% (95% CI 0.07-0.28, I^2^ = 87%, n=113/542) (19, 21, 33). The pooled incidence of chronic GVHD was 36.2% (95% CI 0.27-0.47, I^2^ = 84%, n=400/877) (19, 21, 23, 25, 34, 35, 37). The pooled PFS, RR, and NRM was 46.6% (95% CI 0.32-0.61, I^2^ = 91%, n=280/866), 29% (95% CI 0.21-0.38, I^2^ = 77%, n=294/866), and 20.3% (95% CI 0.18-0.30, I^2^ = 83%, n=227/809), respectively (19, 21, 23, 25, 34, 35, 37) (**Tables 1**, **2**).

## Discussion

Allogeneic hematopoietic stem cell transplantation is a potentially curative therapy for malignant and non-malignant hematologic disorders (4, 38). The mismatched unrelated donor is utilized in clinical settings as an alternate donor strategy for potential recipients of HSCT who lack an HLA-matched donor or haploidentical family donor. Recent improvements in HLA typing, GVHD prophylaxis including TCD and PT-Cy, and supportive care have improved survival and transplant-related mortality in MMUD HSCT recipients (11, 12, 39). Our systematic review and meta-analysis, including 19 studies, report outcomes of 3336 patients who underwent HSCT utilizing a mismatched unrelated donor, primarily with one antigen mismatch (88% of the recipients). The one- and three-year OS after MMUD HSCT was 64% and 42%, respectively. Historically, MMUD HSCT has been associated with higher rates of GVHD and NRM (40); however, our analysis showed an improvement in acute GVHD rates with grade III-IV acute GVHD reported in 15% of MMUD HSCT recipients. The incidence of grade III-IV GVHD in the matched unrelated donor and haploidentical HSCT is 14% and 16% respectively, which is comparable to the incidence of severe GVHD in mismatched unrelated donor HSCT (41).

Historically, many studies have reported poor outcomes of MMUD HSCT compared to matched donors, even with a single allele mismatch. However, these results could have been related to the GVHD prophylaxis regimens used, as higher GVHD incidence was associated with higher NRM after HSCT (19, 21, 31). Similarly, several studies reported a direct correlation between higher NRM and degree of HLA mismatches (38, 40, 42, 43). Kasamon et al. showed that two or more mismatched HLA loci or HLA-C mismatch did not increase the incidence of graft failure or grade III-IV acute GVHD when PT-Cy- based GVHD prophylaxis was used, suggestive of an important role of PT-Cy-based GVHD prophylaxis in MMUD HSCT (35). In our review, analysis based on the number and type of allele mismatching was not done due to the paucity of data; however, most patients included in this meta-analysis had one (88%) or two (11%) antigen HLA-mismatch. The graft source was bone marrow in 19% and peripheral blood stem cells in 81% of the patients. PBSCs are used due to ease of collection and faster engraftment of stem cells. Several studies have reported comparable survival between BM and PBSC with a lower rate of chronic GVHD with BM transplant and a lower relapse rate with PBSC (7, 44, 45). Better quality of life has been reported with bone marrow graft source (46).

The pooled incidence of relapse was 27% in our meta-analysis. Comparing relapse rates across these studies given is challenging given the heterogeneity of underlying hematologic malignancies, conditioning regimens, graft source, and GVHD prophylaxis. A recent study has reported a relapse rate of 31% for HLA-matched donor HSCT with PT-Cy-based GVHD prophylaxis (47). In a Center for International Blood and Marrow Transplant Research (CIBMTR) analysis, 2-year disease-free survival (55% vs. 41%) and OS (67% vs. 54%) were higher with MUD as compared to haplo HCT among RIC recipients (48). At the same time, there were no differences in relapse, non-relapse mortality, disease-free, and OS between MUD and haplo HCT with MAC (48). Bachanova et al. and Saraceni et al. reported similar relapse risk but higher NRM with MMUD than other donors (22, 30).

The optimum conditioning regimen before MMUD is not known. As the conditioning regimen plays a crucial role in HSCT outcomes, seven studies in our analysis showed slightly improved survival in patients treated with reduced-intensity conditioning with a one-year OS of 67% and NRM of 20% as compared to a one-year OS of 64% and NRM of 23% in all patients. Higher intensity regimens led to lower relapse risk but resulted in higher NRM. Additional factors that may intersect with conditioning regimen intensity include age, recipient donor chimerism, and the presence of residual host antigen-presenting cells (25). In a study by Robin et al., higher NRM was reported with MAC conditioning (49). Similarly, Brissot et al. show that RIC was associated with a better OS than MAC (28).

Treatment modalities to mitigate GVHD are essential in improving outcomes related to MMUD HSCT. The addition of ATG to standard cyclosporine and methotrexate for GVHD prophylaxis showed favorable results and a low incidence of severe acute GVHD (26). Kasamon et al. reported that GVHD prophylaxis with PT-Cy based regimen resulted in no incidence of graft failure or acute GVHD grade III-IV (35). Similarly, Al-Malki et al. reported that survival after MMUD remained poor in patients receiving calcineurin inhibitor (CNI)-based GVHD prophylaxis (31). Although a direct comparison among studies using TCD versus PT-Cy-based GVHD prophylaxis was difficult, the best outcomes were reported by four studies using PT-Cy-based GVHD prophylaxis compared to fifteen studies that used non-PT-Cy-based GVHD prophylaxis regimens, with a 1-year OS survival of 82% (vs. 56%), PFS of 67% (vs 41.5%), RR of 24% (vs. 27%), NRM of 9% (vs. 26%), grade III-IV acute GVHD incidence of 18% (vs. 15%), and chronic GVHD rate of 30.5% (vs. 44%). A recent study suggested improved GVHD in Haplo HCT patients with BM stem cells compared to PBSC when PT-Cy was used for prophylaxis with similar NRM and OS (50).

To our knowledge, this is the first meta-analysis to determine the outcomes after MMUD HSCT. Our meta-analysis has several limitations. Studies included in the meta-analysis were heterogeneous. Most of the included studies were conducted without randomization and blinding and did not have comparator arms. The study compared outcomes among different hematological malignancies with varying disease biology that could affect results, particularly the relapse rates. The data was insufficient to perform a separate analysis based on the type of hematologic disease. We reported outcomes for reduced-intensity conditioning and PT-Cy-based and non-PT-Cy-based GVHD prophylaxis separately, but data is not available for a head-to-head comparison.

## Conclusion

Mismatched unrelated donor HSCT has demonstrated favorable outcomes with an acceptable toxicity profile. MMUD HSCT outcomes using reduced-intensity conditioning and post-transplant cyclophosphamide-based GVHD prophylaxis regimen are comparable to the mismatched related donor (haploidentical) HSCT outcomes. Our findings suggest a single-antigen mismatched unrelated donor HSCT should be utilized in patients lacking an HLA-matched donor or haploidentical family donor. This strategy may expand HSCT access, especially for racial and ethnic minority populations currently underrepresented in the donor registries.

## Data availability statement

The original contributions presented in the study are included in the article/**Supplementary Material**. Further inquiries can be directed to the corresponding author.

## Author contributions

All authors contributed to the manuscript and fulfilled criteria per the uniform requirements set forth by the International Committee of Medical Journal Editors (ICJME) guidelines. All authors have reviewed and approved the final version of the manuscript.

## Conflict of Interest

SA has speaking, consulting and advisory role, and research funding from Incyte and Therakos. JM has speaking, consulting and advisory role in Kite, Juno Therapeutics, Allovir, Magenta Therapeutics, EcoR1 Capital, and has research funding from Novartis, Fresenius Biotech, Astellas Pharma, Bellicum Pharmaceuticals, Gamida Cell, Pluristem Therapeutics, Kite and AlloVir.

The remaining authors declare that the research was conducted in the absence of any commercial or financial relationships that could be construed as a potential conflict of interest.

## Publisher’s note

All claims expressed in this article are solely those of the authors and do not necessarily represent those of their affiliated organizations, or those of the publisher, the editors and the reviewers. Any product that may be evaluated in this article, or claim that may be made by its manufacturer, is not guaranteed or endorsed by the publisher.
