# Supplementary material for: Outcomes with mismatched unrelated donor allogeneic hematopoietic stem cell transplantation in adults: A systematic review and meta-analysis
Source: Front Oncol. 2022 Oct 6;12:1005042. doi: 10.3389/fonc.2022.1005042 (PMC9583270; doi:10.3389/fonc.2022.1005042)
Supplement: Supplementary file 1 [file Table_1.docx]

Supplementary Material

**Supplementary Table 1: Excluded studies with reason and DOI**

| **No.** | **Author Name** | **Reason For Exclusion** | **DOI/Link/Reference** |
| --- | --- | --- | --- |
| 1 | Ringden | Review Article | https://link.springer.com/article/10.1007/BF02990940 |
| 2 | Ringden | Irrelevant | https://pubmed.ncbi.nlm.nih.gov/10397713/ |
| 3 | Spyridondis | Irrelevant | DOI: 10.1182/blood-2005-01-0196 |
| 4 | Sun | Irrelevant | DOI: 10.3324/haematol.2015.140509 |
| 5 | Topolsky | No data | https://pubmed.ncbi.nlm.nih.gov/8722353/ |
| 6 | Vigoroux | Irrelevant | DOI: 10.3109/10428194.2011.604754 |
| 7 | Watkins | Abstract | https://www.cochranelibrary.com/central/doi/10.1002/central/CN01453357/full |
| 8 | Yu | No MMUD data | DOI: 10.1007/s00277-020-04199-9 |
| 9 | Zheng | No MMUD data | DOI: 10.1016/j.bbmt.2013.09.008 |
| 10 | Qian | No mismatched data | DOI: 10.1186/s13045-017-0469-0 |
| 11 | Remberger | No mismatched data | DOI: 10.1038/sj.bmt.1701991 |
| 12 | Ringden | no mismatched data | https://pubmed.ncbi.nlm.nih.gov/9712503/ |
| 13 | Spellman | No relevant data | DOI: 10.1016/j.bbmt.2009.03.018 |
| 14 | Staba | Pediatric study | DOI: 10.1056/NEJMoa032613 |
| 15 | Sun | Chinese | DOI: 10.3760/cma.j.issn.0253-2727.2014.08.002 |
| 16 | Sun | Pediatric | DOI: 10.1016/j.bbmt.2019.03.028 |
| 17 | Untereger | No mismatched data | DOI: 10.1038/bmt.2012.242 |
| 18 | Uzunel | No mismatched data | DOI: 10.1097/01.tp.0000233865.20232.51 |
| 19 | Vander Lugt | Pediatric | DOI: 10.1182/bloodadvances.2020001940 |
| 20 | Venstrom | No mismatched data | DOI: 10.1182/blood-2009-08-236943 |
| 21 | Wadhwa | Case Report | DOI: 10.1016/s0145-2126(02)00090-5 |
| 22 | Walter | No mismatched data | DOI: 10.1038/leu.2010.102 |
| 23 | Wang | No mismatched data | DOI: 10.1007/s12185-013-1442-5 |
| 24 | Wang | No mismatched data | DOI: 10.1016/j.bbmt.2017.03.032 |
| 25 | Wang | Irrelevant | DOI: 10.1016/j.bbmt.2018.05.027 |
| 26 | Weisdorf | No mismatched data | DOI: 10.1053/bbmt.2002.v8.pm12014810 |
| 27 | Weismann | Case Report | DOI: 10.1038/sj.bmt.1704191 |
| 28 | Woolfrey | No mismatched data | DOI: 10.1016/j.bbmt.2010.03.024 |
| 29 | Yabe | Irrelevant | DOI: 10.1111/j.1365-2141.2006.06128.x |
| 30 | Yanada | No mismatched data | DOI: 10.1111/ejh.12723 |
| 31 | Yanada | No mismatched data | DOI: 10.1038/bmt.2012.159 |
| 32 | Yang | No mismatched data | DOI: 10.1038/s41409-018-0382-3 |
| 33 | Yu | No mismatched data | DOI: 10.1007/s00277-020-04199-9 |
| 34 | Yu | No mismatched data | DOI: 10.1097/md.0000000000002973 |
| 35 | Zhang | Combined data | DOI: 10.1016/j.bbmt.2019.03.020 |
| 36 | Zheng | Focuses on GVHD | DOI: 10.1038/bmt.2016.182 |
| 37 | Zohren | Focuses on GVHD | DOI: 10.1038/bmt.2010.167 |
